# The effect of a nurse-led low carbohydrate regimen on anthropometric and laboratory parameters of patients with metabolic syndrome: a quasi-experimental study

**DOI:** 10.3389/fpubh.2024.1415916

**Published:** 2024-07-17

**Authors:** Mohammed Faris Abdulghani, Sadeq Al-Fayyadh

**Affiliations:** Nursing College, University of Baghdad, Baghdad, Iraq

**Keywords:** metabolic syndrome, low carbohydrate diet, nurse-led intervention, anthropometric parameters, laboratory parameters, quasi-experimental study

## Abstract

**Introduction:**

Metabolic syndrome is a global health concern. It is a condition that includes a cluster of various risk factors for type 2 diabetes and cardiovascular disease. This quasi-experimental study investigates the effect of a nurse-led low-carbohydrate regimen on anthropometric and laboratory parameters in metabolic syndrome patients.

**Methods:**

The study used a quasi-experimental design conducted at the University of Mosul; 128 participants meeting the metabolic syndrome criteria were recruited and divided into the intervention and control groups. The intervention group received personalized counseling and support in implementing a low-carb regime, while the control group received standard advice. The study participants were assessed by anthropometry, and laboratory parameters were evaluated pre- and post-intervention. Statistical data analysis was conducted using IBM-SPSS 27, including chi-square, Fisher’s exact test, *t*-tests, and the Mcnemar test, which were performed to compare the changes within and between groups.

**Results:**

The mean age of the participants in the intervention and control groups was 50.72 ± 6.43 years and 49.14 ± 6.89 years, respectively. Compared to the control group, the intervention group experienced a significant positive reduction in anthropometric measures and laboratory parameters, including weight, body mass index (BMI), waist circumference, lipid profiles, and HbA1c.

**Conclusion:**

A tangible effect of nurse-led interventions based on low-carbohydrate regimens in managing metabolic syndrome was empirically authenticated. Positive changes were observed in the intervention group regarding anthropometric measures and laboratory parameters. However, future research may require a larger sample size and a longer follow-up to confirm these effects and evaluate long-term metabolic impacts.

## 1 Introduction

Metabolic syndrome is a cluster of diseases that may raise the risk of type 2 diabetes and cardiovascular disease (1). The main components of metabolic syndrome include insulin resistance, dyslipidemia, abdominal obesity, and high blood pressure (2). Adult Treatment Panel III (ATP III) guidelines defined metabolic syndrome (Mets) as being affected by three or more of the following criteria: (1) waist circumference (WC) more (102 cm) for men and more (88 cm) for females women; (2) serum triglycerides equal to or more than (150 mg/dL.); (3) high-density lipoprotein (HDL) levels (<50 mg/dL) in women and (<40 mg/dL) in men; (4) fasting blood glucose levels (>100 mg/dL) or use of antidiabetic medications (insulin or oral agents); (5) finally, increases blood pressure both in systolic at (130 mmHg) or higher and in diastolic (85 mmHg) or more (3).

Mets are generally associated with an increased probability of many chronic diseases, mainly cardiovascular disorders (CVD) and diabetes, which are also associated with increased cardiovascular mortality (4, 5). The incidences of Metabolic syndrome have increased rapidly in the last 30 years between different sociodemographic groups (6). Iraq is one of the largest Arab Gulf countries in the Middle East region; overall, 41.3% of Iraqi students suffer from metabolic syndrome, with 66.9% of women suffering from metabolic syndrome (7). Mets are widespread and appear in all areas of the world, in people with low physical activity and high-calorie intake (8). An unhealthy diet can increase the risk of metabolic syndrome, especially in older people. Those with healthy diets have a lower risk of obesity and high blood pressure. Thus, improving diet can help manage Mets, providing critical insights for future research-based recommendations (9).

Low-carbohydrate diets, known since the 1860s, are now commonly used for weight loss and disease treatment. However, the specific contents of these diets vary, with the only consistent element being a decrease in the amount of carbohydrates consumed (10).

A recent study classified the regimen according to total daily carbohydrate consumption. The first category is very low carbohydrate (VLC), representing less than 10% carbohydrates or 20–50 g/day. The second category is low carbohydrate, which means less than 26% carbohydrates or less than 130 gm/day. The third category is moderate carbohydrates, representing 26–44%. The fourth category is high carbohydrate, which means 45% or more of carbohydrates out of the total calorie intake (11). A low-carb regimen reduces carbohydrate intake while increasing protein and healthy fat (12). This method has been commonly used in recent years to control or manage the symptoms of diseases such as diabetes, obesity, and metabolic syndrome by improving insulin sensitivity and weight loss; however, this method’s effect is still not empirically confirmed (13).

Low-carbohydrate diets could significantly improve waist circumflex, blood pressure level, cholesterol, triglycerides, and HbA1. All may positively reduce cardiovascular disease and DM2 risk in people with metabolic syndrome. Therefore, managing Mets with a low carbohydrate diet has an outstanding reputation as an excellent approach to managing metabolic syndrome (14–16). Furthermore, studies have shown that LCD can improve indicators of metabolic syndromes, such as blood pressure control, blood sugar control, and maintaining optimal cholesterol level(s) (1, 17). In addition, a low carbohydrate diet is beneficial for weight loss and enhancing insulin sensitivity, which can reduce the risk of Mets and problems associated with it (18).

A large meta-analysis verified that low-carbohydrate diets significantly influenced cardiovascular disorders (19). A meta-analysis study involving RCTs illustrated that low-carbohydrate diets are associated with a significant loss of weight and reduced levels of low-density lipoprotein cholesterol (LDL) (20). A considerable randomized controlled trial found that the low-carbohydrate diet led to weight, blood pressure, triglycerides, and HDL cholesterol levels reduction compared to the low-fat diet (21). Of equal importance, a recent randomized controlled trial found that the low carbohydrate diet has the ability to enhance glycaemic control, weight loss, and cardiovascular disorder in contrast to the plate method diet (22). Examining the effect of the low carbohydrate regime on individuals with metabolic syndrome is significant, as it reveals prospective dietary management techniques for this health problem (23). The current study’s findings may improve treatment options, outcomes, and consequences for people with metabolic syndrome.

Several dietary strategies, such as a low-carbohydrate diet, have been proposed to improve metabolic markers in patients with Mets. However, the effectiveness of a low-carbohydrate regimen in improving these markers remains uncertain, with much empirical evidence (24). Although some studies have found significant findings, others have not found differences between a low-carbohydrate diet and other types of diet (25, 26). Therefore, compared to alternative diets, determining the effect of a reduced-carbohydrate diet on metabolic markers in patients with metabolic syndrome requires further investigation. This will help clarify this diet approach’s potential benefits and risks in developing a guideline for treating metabolic syndrome based on evidence and identify the optimal diet approach for these patients.

Furthermore, there is an absence of evidence concerning the effectiveness of such a regimen when managed by nurses. Understanding the role of nurses in the direction of dietary intervention is crucial; it offers significant information on the benefits of low-carbohydrate diets on metabolic health but does not specifically address the impact of nurse-led initiatives. Therefore, this study aims to examine the effect of a nurse-led low carbohydrate regimen on anthropometric and laboratory parameters of patients with metabolic syndrome, which could offer new insights to improve metabolic health through nursing intervention. Furthermore, there are no Iraqi studies on this subject, which underscores the novelty and significance of such research in the local context. The purpose of this study was to investigate the impact of low-carbohydrate diets led by nurses on Iraqi patients with metabolic syndrome. The primary outcomes measured were improvements in anthropometric measures such as weight, BMI, and waist circumference and in laboratory parameters, including lipid profiles and HbA1c. This study could provide new insights into improving metabolic health through nursing intervention and potentially fill a significant gap in understanding metabolic syndrome’s global and local management.

## 2 Materials and methods

### 2.1 Type of study and setting

The study design employed in this research was a non-randomized quasi-experimental design with a pre-test-post-test control group. The research was conducted at Mosul University in the Nineveh province of northern Iraq. Mosul University is an essential educational and research institution in Mosul, Iraq. Founded in 1967, it is Iraq’s largest and most respected university, behind the University of Baghdad. The University offers bachelor’s, master’s, and doctoral degrees in various fields, such as medicine, engineering, humanities, arts, science, nursing, and agriculture. Employees, students, and outpatient clients of the University Research Hospital at Mosul University constituted the participants of this study.

### 2.2 Sample size and sampling

All patients meeting the inclusion criteria were selected and examined using purposive sampling. The inclusion criteria consisted of the following: having metabolic syndrome according to NCEP ATP-III criteria, including abdominal obesity (waist circumference > 102 cm in men, >88 cm in women), high triglycerides (serum level ≥ 150 mg/dL), low HDL cholesterol (HDL <40 mg/dL in men, <50 mg/dL in women), elevated blood pressure (≥130/85 mmHg), and elevated fasting glucose (≥100 mg/dL); maintaining stable health conditions, without acute medical events or hospitalization during the defined period of 3 months before the study; and being free from chronic diseases such as cardiovascular diseases, chronic kidney disease, chronic liver diseases, diabetes, chronic pancreatitis, or cancer.

**Figure 1:**
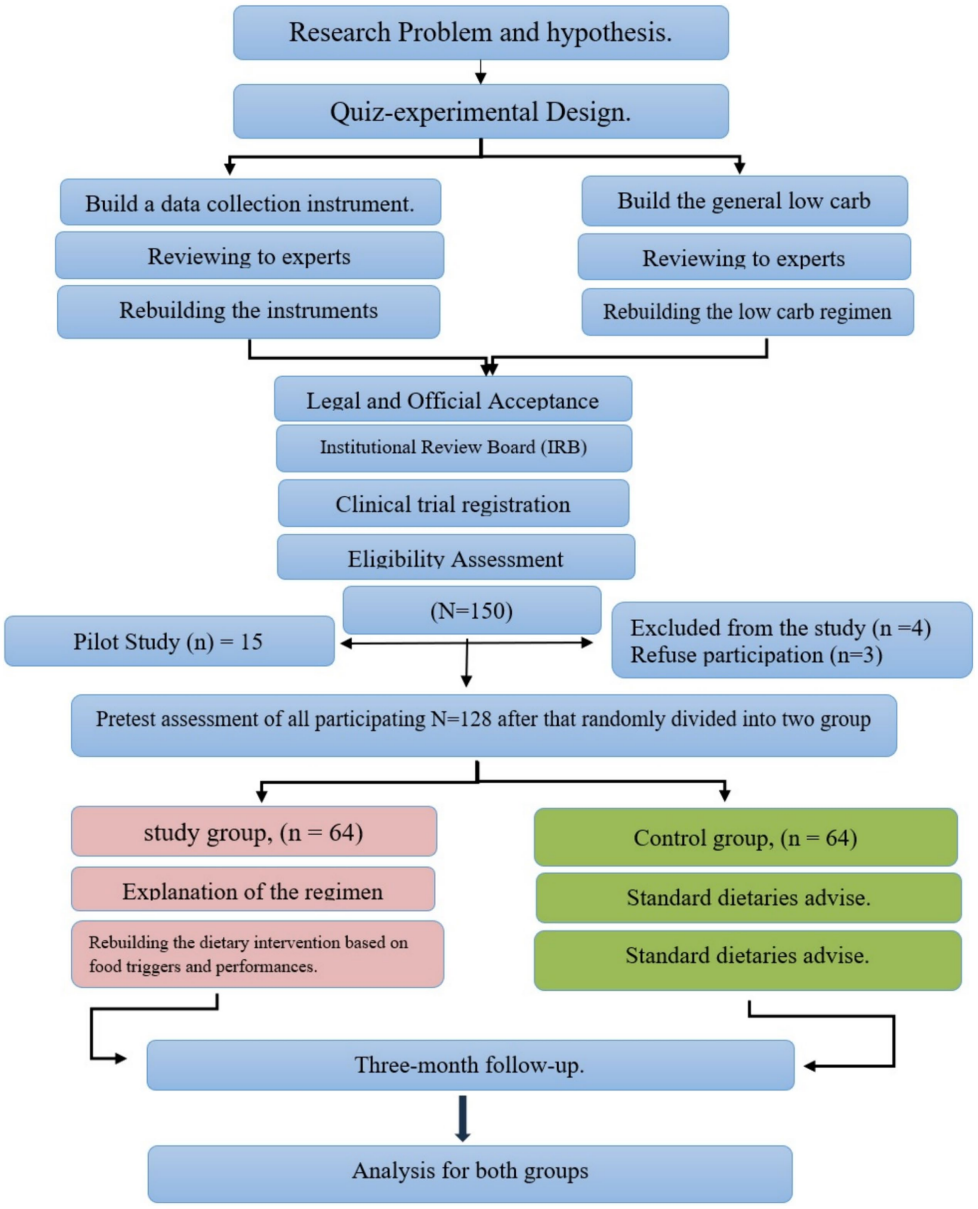
Study protocol algorithm.

Exclusion criteria included the use of certain medications such as antidiabetic, antihypertensive, obesity, and hyperlipidemia drugs, as well as participation in other diet and exercise programs within the last 6 months. Eligible patients were then divided into intervention and control groups, with participants stratified based on age, sex, education level, and clinical characteristics (e.g., baseline levels of metabolic syndrome parameters) (Figure 1).

The sample size was estimated according to the *A-Priori* sample size for the *T*-test: Cohen’s *d* (effect size), desired power, and alpha level (probability level). The expected effect size is 0.5 (Cohen *d*), which indicates a medium effect size. The preferred level of statistical power is 0.8 (80%), which is usually used in social sciences to minimize the chance of Type II error. The probability level (alpha) is fixed at 0.05, indicating the probability of 5% of Type I errors. Finally, 128 patients were included in the study.

### 2.3 Data collection tools

A comprehensive questionnaire was used to gather data, including three main subsections: the demographic and clinical information registration form, anthropometric measurements, and laboratory parameters.

### 2.4 Demographic and clinical information

The demographic and clinical information documentation form involved several elements, including the patient’s age, gender, residence, education level, and Job-related situation. It also investigated the patient’s medical-surgical history, detailed records of medication intake, and family health history. Additionally, the form captured information related to eating habits and lifestyle.

### 2.5 Anthropometric measurements

Including weight, height, BMI, waist and hip circumference, and waist-to-hip ratio, were collected from the patients.

Weight and height measurement: The height and weight are estimated to be 0.1 kg and 0.5 cm, respectively, and the BMI is calculated. The weight was measured by SECA and manufactured for UNICEF using Australia’s technology (SN: 3101615).

Waist circumference and hip circumflex (HC): WHO guidelines were used to measure the WC and HC using a non-stretchable tape measure SECA-201 (SECA, Hamburg, Germany).

Skinfold thickness: It is measured at the triceps area using Holtain’s skinfold calipers (Holtain Ltd., Crymych, United Kingdom).

### 2.6 Measurement of biochemical parameters

Included lipid profiles [S. cholesterol, S. High-Density Lipoprotein (HDL), S. Low-Density Lipoprotein (LDL), S. Triglyceride], which were estimated by automated clinical analyzer; ARCHITECT PLUS model C4000 manufactured by Abbott SN C460240 made in the USA manufactory year May 2, 2021, and average blood sugar levels for 3 months through the Hemoglobin A1c (HbA1c) by using the Quo-Lab^®^ HbA1c Analyzer from EKF Diagnostics Serial Number: 023894, Country of Manufacture Germany.

The face and content validity of the study instruments were assessed through submission to a panel of (15) experts representing different specialties related to the study objectives. The reliability test was done to assess the errors in the measurement technique; therefore, each instrument used in the current study was assessed by statistical analysis. Correlation coefficients were computed to measure the reliability of the study tools throughout the application of the Test–retest reliability coefficients (also called coefficients of stability), which was of high reliability in average at r value in the current study for continuous variables including anthropometric measurement (height, weight, waist circumflex, hip circumflex), blood pressure and biochemical test include lipid profile and HbA1c. This means the study tools are stable and reliable. This involves measuring the same (15) participants at two different points and then calculating the correlation coefficient between the two sets of measurements. The researchers ensure that no interventions or treatments are applied between these two points that could affect the measurements. The Pearson correlation coefficient is between −1 and +1. A coefficient near +1 indicates high reliability, meaning the measurements are consistent over time.

### 2.7 Data collection

Initially, the researcher obtains approval of the research protocol from the adult nursing department at the Bagdad nursing faculty after introducing the scientific plan and methods and getting permission to start the study, then gets official approval from the Institutional Review Board (IRB)-Scientific Committee of the Nursing College at the University of Baghdad. The researchers proceeded to the research site at Mosul University and commenced sampling. Upon obtaining written informed consent from each patient, demographic and clinical information questionnaires were completed. Additionally, all metabolic parameters, including anthropometric data (such as BMI, waist circumference, triceps skinfolds, and hip circumference), blood pressure measurements, and blood samples (lipid profile, HbA1c), were collected from both the study and control groups at baseline. Then, the researcher used the stratification process involving three categories: age, gender, and education level. Following stratification, a computer-generated random numbers table was applied within all subgroups to allocate participants to the intervention or control group according to the study’s entry criteria: intervention and control. This method is designed to minimize selection bias and confounding variables between the two groups.

Patients assigned to the intervention group underwent a three-month low-carbohydrate diet regimen. In the low-carb diet, consumption of high-carbohydrate foods such as bread, pasta, biscuits, cakes, and sugar was restricted. These items were replaced with low-carb alternatives like spinach, cauliflower, and other fiber- and protein-rich foods such as eggs, meat, cheese, nuts, fish, and poultry. In this system, the percentage of carbohydrates did not exceed 30% of the total calories, which equates to a maximum of 130 g. These carbohydrates were sourced from healthy options.

Participants in this group also received comprehensive individual education regarding low-carbohydrate diets. The researchers dedicated 5 days a week, from 8:30 a.m. to 1:30 p.m., to collect data. Each data collection interview took approximately 30–45 min per patient. The educational content is detailed in Table 1.

**Table 1:** The educational content of adherence to a low-carbohydrate diet.

Training	Content
Patient education and counseling	
Initial consultation	Explaining the concept of a low-carbohydrate diet, its benefits, potential challenges, and the importance of adherence in treating metabolic syndrome underscores the significance of monitoring, supporting, and encouraging patients to achieve their goals.
Diet training	A comprehensive explanation of diet, explaining nutritional principles, including calories, macronutrients, meal timing, supplements, and dietary guidelines, explaining how to get the right nutrients and avoid unhealthy foods.
Individual meal planning	
Nutritional assessment	Detailed nutritional assessments allow us to comprehensively understand each patient’s health habits, preferences, and dietary needs.
Identification of food triggers	Identifying the specific social, emotional, situational, cognitive, and physiological factors that trigger overeating helps address barriers to participants’ achieving their goals by using the Trigger Food Questionnaire.
Management of food triggers	We implement teaching strategies aimed at managing identified triggers, employing a problem-solving approach to develop healthy coping mechanisms for emotional stress instead of resorting to food. This includes teaching participants to plan for social situations where unhealthy foods may be present, raising awareness of situational triggers and cognitive behaviors, and devising plans to address them. For instance, we encourage finding alternative activities to eat when bored and challenging negative thinking patterns that lead to overeating. Additionally, participants are guided to focus on the eating experience, recognizing hunger and fullness cues, and slowing down to savor the taste and texture of food, promoting conscious eating practices.
Cooking and shopping tips	Researchers offer practical advice on food preparation, cooking methods, and shopping for low-carb foods to facilitate easy adherence to dietary goals.

Throughout the intervention period, researchers provided educational materials, booklets, and digital resources to patients, offering guidance on foods based on the glycemic index. Additionally, personal feedback sessions were scheduled for each patient to allow them to express their views. Alternatively, a structured follow-up plan was implemented by the researchers, involving weekly in-person phone meetings to monitor progress, adjust meal plans, and address any issues or concerns. Additionally, to ensure the intervention’s proper implementation, some calls included not only the patient but also a family member, aimed at reinforcing the patient’s adherence to the intervention. This approach facilitated control within the intervention group and ensured high compliance. Participants were also encouraged to reach out to the researcher between appointments for any questions or concerns regarding their diet or general health. The educational intervention was conducted from 1st July to 31st October 2023.

No intervention was conducted in the control group. Instead, participants in this group maintained their regular eating habits, continued their usual diet, and received standard care.

Three months later, the same series of parameters were re-measured, including anthropometric, blood tests, blood pressure measurements, food triggers, and signs and symptoms of patients with metabolic syndrome in both groups. This step is crucial for comparing pre- and post-intervention data within each group and between the study and control groups.

### 2.8 Data analysis

Statistical data analysis was conducted using IBM-SPSS27. A descriptive method was applied as Mean ± SD, or frequency (percentage) was computed. A Chi-square test was used to examine the parameters of metabolic syndrome patients. Mcnemar test for categorical variables in paired proportions. Fisher’s exact test for categorical variables, one paired t-test, applies to comparing two means from two related groups. An independent t-test compares the means between two unrelated (independent) groups for continuous variables following a normal distribution.

### 2.9 Ethical considerations

This research was conducted after obtaining the institutional review board agreement numbered Ref: 1540 on May 8th, 2023, from Baghdad University. The clinical trial was formally registered with the Iranian clinical trial registry. The clinical trial code is IRCT20231002059587N1. A thorough explanation was provided regarding the study’s objectives and the potential utilization of its findings for the subjects involved. Written consent was obtained from the participants for their inclusion in the study, and they were granted the freedom to withdraw at any time. Participants were assured of the confidentiality of their information. Additionally, they were informed that their treatment process would remain unchanged regardless of their decision to participate, following Consolidated Standards of Reporting Trials guidelines recommended by the EQUATOR Network.

## 3 Results

The results of the present study showed that participants in both intervention and control groups had similar mean ages, balanced gender and education levels distribution, and predominantly urban residence. For all these variables, no significant differences were found between the control and intervention groups. While there was no disparity in SBP between groups (*p* = 0.793), the intervention group had a lower mean DBP (*p* = 0.004). No significant differences were observed in various health indicators and histories (*p* > 0.05) except for smoking, where the intervention group had higher rates and longer duration (*p* < 0.001). Additionally, the intervention group reported more frequent fast-food consumption (*p* = 0.007) and different reasons for discontinuing previous dietary regimens (*p* = 0.003), though medication usage for metabolic syndrome did not differ significantly between groups (*p* > 0.05) (Table 2).

**Table 2:** Comparison of demographic characteristics, medical history, dietary habits, and lifestyle of participants between intervention groups and control groups.

Group	Intervention (*n* = 64)		Control (*n* = 64)		Statistical test	*p*-value
Variable						
	Mean	*SD*	Mean	*SD*		
Age (year)	50.72	6.43	49.14	6.89	*t* = 1.34	0.183
Current SBP (mmHg)	143.83	5.33	144.12	7.31	*t* = −0.26	0.793
Current DBP (mmHg)	90.59	4.29	93.44	6.56	*t* = −2.90	0.004
	*N*	%	*N*	%		
Age group/year					*χ*^2^ = 1.43	0.921
28–33	1	1.6	1	1.6		
34–39	6	9.4	6	9.4		
40–45	7	10.9	11	17.1		
46–51	16	25.0	17	26.5		
52–57	27	42.2	24	37.4		
58–63	7	10.9	5	7.7		
Total	64	100.0	64	100.0		
Sex						
Male	32	50.0	31	48.4	*χ*^2^ = 0.03	0.860
Female	32	50.0	33	51.6		
Place of residence						
Rural	3	4.7	5	7.8	*χ*^2^ = 0.53	0.465
Urban	61	95.3	59	92.2		
Levels of education	Number	%	Number	%		
Able to read and write	1	1.56	1	1.56	Fisher’s exact test = 56	0.336
Primary school	2	3.13	2	3.13		
Secondary school	2	2.50	3	4.69		
Preparatory school	8	40.63	7	10.94		
Undergraduate	26	10.94	20	31.25		
Postgraduate Diploma	7	23.44	5	7.81		
Postgraduate Master	15	4.69	20	9.38		
Postgraduate PhD	3	1.56	6	1.56		
Occupation						
Employed	58	90.6	51	79.7	*χ*^2^ = 3.03	0.082
Unemployed	6	9.4	13	20.3		
History of HTN						
Yes	31	48.4	34	53.1	*χ*^2^ = 0.28	0.596
No	33	51.6	30	46.9		
History of surgery						
Yes	11	17.2	20	31.3	*χ*^2^ = 3.45	0.063
No	53	82.2	44	68.8		
Obesity						
Yes	43	67.2	36	56.3	*χ*^2^ = 1.62	0.203
No	21	32.8	28	43.8		
Family history of HLD						
Yes	6	9.4	14	21.9	*χ*^2^ = 3.79	0.051
No	58	90.6	50	78.1		
Family history of HD						
Yes	19	29.7	23	35.9	*χ*^2^ = 0.57	0.451
No	45	70.3	41	64.1		
Family history of DM						
Yes	28	43.8	32	50.0	*χ*^2^ = 0.50	0.479
No	36	56.2	32	50.0		
History of smoking						
Yes	31	48.4	12	18.8	*χ*^2^ = 12.64	<0.001
No	33	51.6	52	81.2		
Smoking duration						
≤5 years	4	6.2	1	1.6	*χ*^2^ = 12.78	0.002
>5 years	27	42.2	11	17.2		
No smoking	33	51.6	52	81.2		
Passive smoking						
Yes	37	57.8	42	65.6	*χ*^2^ = 0.83	0.363
No	27	42.2	22	34.4		
Alcohol consumption						
Yes	0	0.0	4	6.3	Fisher’s exact test = 4.13	0.060
No	64	100.0	60	93.7		
Number of meals per day						
1	9	14.0	15	23.4	*χ*^2^ = 5.03	0.169
2	21	32.8	26	40.6		
3	28	43.8	21	32.8		
4	6	9.4	2	3.2		
Number of fast-food meals per week						
1	9	14.1	9	14.1	*χ*^2^ = 13.94	0.007
2	15	23.4	20	31.3		
3	6	9.4	13	20.3		
4	2	3.1	8	12.4		
>4	32	50.0	14	21.9		
Previous dietary regimens						
Yes	18	28.1	22	34.4	*χ*^2^ = 0.58	0.446
No	46	71.9	42	65.6		
Type of previous diet:						
Low-carbohydrate	0	0.0	1	1.6	Fisher’s exact test = 9.78	0.094
Keto	0	0.0	4	6.3		
Low calories	7	10.9	10	15.6		
Low-fat	5	7.8	5	7.8		
Metatherian	2	3.1	2	3.1		
Others	4	6.3	0	0.0		
None	46	71.9	42	65.6		
Reason for stopping diet regimens:						
Difficulty adhering	2	3.1	13	20.3	*χ*^2^ = 13.12	0.003
Lack of results	8	12.5	6	9.4		
Others	8	12.5	3	4.7		
None	46	71.9	42	65.6		
Sleep at night/(hours)						
2	0	0.0	1	1.6	Fisher’s exact test = 21.99	<0.001
3	3	4.7	10	15.6		
4	0	0.0	7	10.9		
5	0	0.0	4	6.3		
6	36	56.3	20	31.3		
7	16	25.0	10	15.6		
8	9	14.0	12	18.7		
History of using medication to treat metabolic syndrome						
Yes	2	3.1	6	9.4	*χ*^2^ = 2.13	0.137
No	62	96.9	58	90.6		

SD, standard deviation; *t*, independent *t-*test, *χ*^2^, Chi square. SBP, systolic blood pressure; DBP, diastolic blood pressure; HTN, hypertension; HLD, hyperlipidemia; HD, heart disease; DM, diabetes mellitus.

Table 3 results indicate that initially, there was no statistically significant variance in the BMI scores between the intervention and control groups. However, following the intervention, the BMI scores of patients in the intervention group exhibited a significant decrease compared to those in the control group (*p* < 0.001). Before the intervention, there were no significant differences between groups in waist and hip circumferences and waist-hip ratio. Post-intervention, waist circumference significantly decreased in the intervention group compared to the control (*p* < 0.001), with no differences in hip circumference and waist-hip ratio. Additionally, within-group analysis reveals a significant decrease in triceps skinfold in the intervention group after intervention (*p* < 0.001). The atherogenic index significantly decreased after intervention in the intervention group compared to the control (*p* < 0.001), with no significant changes in the control group. There was no significant difference in obesity type before intervention between groups, but after intervention, gynoid obesity significantly increased in the intervention group compared to the control (*p* = 0.001). Within-group changes in obesity type were not significant for either group.

**Table 3:** Comparison of the anthropometric measurements between study and control groups, pre and post intervention.

Group			Intervention (*n* = 64)		Control (*n* = 64)		Independent *t*-test	*P*-value
Variable								
			Mean	*SD*	Mean	*SD*		
Body Mass Index		Before	30.27	3.78	30.28	3.92	−0.009	0.993
		After	27.54	3.74	30.56	4.03	16.964	<0.001
Paired *t*-test			−15.18		0.048			
*P*-value			<0.001		0.961			
Waist circumflex (cm)		Before	105.55	7.24	105.38	7.56	0.13	0.896
		After	100.21	7.31	105.42	7.41	−4.00	<0.001
Paired *t*-test			−20.13		0.47			
*p-*value			<0.001		0.638			
Hip circumflex (cm)		Before	102.68	9.50	103.10	9.14	−0.26	0.798
		After	101.62	9.66	103.35	9.10	−1.04	0.301
Paired *t*-test			−4.88		2.28			
*P-*value			<0.001		0.026			
Waist Hip Ratio		Before	1.04	0.15	1.04	0.14	0.09	0.933
		After	1.05	0.15	1.03	0.14	0.72	0.476
Paired *t*-test			0.38		−1.81			
*P-*value			0.704		0.076			
Triceps skin fold (cm)		Before	23.74	7.61	23.59	5.70	0.12	0.901
		After	20.94	5.15	23.63	5.57	−2.84	0.005
Paired *t*-test			−5.84		0.55			
*P-*value			<0.001		0.582			
Atherogenic index		Before	4.71	1.57	5.49	1.29	−3.08	0.003
		After	2.75	1.15	5.48	1.35	−12.37	<0.001
Paired *t*-test			−10.73		−0.21			
*P-*value			<0.001		0.834			
			*N*	%	*N*	%	Fisher’s exact test	*P*-value
Type of obesity	Before	Android	60	93.8	64	100.0	4.13	0.060
		Gynoid	4	6.3	0	0.0		
	After	Android	55	85.9	64	100.0	9.68	0.001
		Gynoid	9	14.1	0	0.0		
Mcnemar test *P*-value			0.267		–			

Table 4 reveals that total cholesterol and triglyceride levels did not significantly differ between groups before the intervention, but post-intervention, they significantly decreased in the intervention group compared to the control (*p* = 0.002). Within-group analysis showed significant decreases in total cholesterol and triglycerides after intervention in the intervention group (*p* < 0.05). HDL and LDL levels significantly differed before and after intervention between groups, with HDL significantly higher in the intervention group post-intervention (*p* < 0.05). However, LDL did not differ significantly between groups. Within the intervention group, HDL increased significantly post-intervention (*p* < 0.001). There were no significant changes in LDL in either group. Before the intervention, FBS and HbA1c levels did not significantly differ between groups. However, post-intervention, they significantly decreased in the intervention group compared to the control, with significant decreases observed within the intervention group post-intervention (*p* < 0.001).

**Table 4:** Comparison of the biochemical tests between study and control groups, before and after the intervention.

Group		Intervention (*n* = 64)		Control (*n* = 64)		Independent *t*-test	*p*-value
Variable							
		Mean	*SD*	Mean	*SD*		
Total cholesterol	Before	213.00	40.16	222.05	29.56	−1.45	0.15
	After	205.78	30.60	222.12	29.13	−3.10	0.002
Paired *t*-test		−2.66		0.20			
*P-*value		0.010		0.842			
HDL	Before	38.65	8.95	34.89	4.92	2.95	0.004
	After	43.18	7.94	35.01	5.11	6.93	<0.001
Paired *t*-test		6.60		1.27			
*P-*value		<0.001		0.210			
LDL	Before	128.69	30.08	142.34	23.58	−2.86	0.005
	After	131.27	25.72	142.36	23.45	−2.55	0.012
Paired *t*-test		1.22		0.07			
*P-*value		0.226		0.946			
Triglyceride	Before	228.29	80.23	224.08	78.25	0.30	0.764
	After	156.62	40.61	223.80	75.24	−6.28	<0.001
Paired *t*-test		−12.71		−0.16			
*P-*value		<0.001		0.871			
Fasting blood sugar	Before	133.93	15.37	131.31	17.17	0.91	0.364
	After	103.98	8.64	130.37	15.81	−11.72	<0.001
Paired *t*-test		−19.66		−1.58			
*P-*value		<0.001		0.119			
HbA1c	Before	6.29	0.54	6.20	0.60	0.91	0.364
	After	5.25	0.30	6.17	0.55	−11.72	<0.001
Paired *t*-test		−19.66		−1.58			
*P-*value		<0.001		0.119			

## 4 Discussion

This study was conducted to determine the effect of a nurse-led low-carbohydrate regimen on anthropometric and laboratory parameters of patients with metabolic syndrome. Based on the results of the present study, the nurse-led low-carbohydrate regime had a significant impact on BMI, reducing waist circumference, triceps skinfold, and atherogenic index in the intervention group compared to the control group. However, no significant effects were observed on hip circumference, and waist-to-hip ratio.

The observed findings can be attributed to several factors. Firstly, the decrease in waist circumference in the intervention group may be explained by the effects of a low-carbohydrate diet on reducing insulin levels, leading to the breakdown of stored fat, particularly in the abdominal region (27). Different dietary patterns, including low-carb diets, may influence body fat distribution, potentially explaining the impact on the type of obesity observed in the intervention group. The reduction in triceps skinfold thickness suggests a decrease in subcutaneous fat (28), possibly due to overall fat loss associated with low-carbohydrate diets. Moreover, low-carbohydrate diets have been linked to improvements in cardiovascular health markers. By reducing carbohydrate intake, these diets may lower triglyceride levels and increase high-density lipoprotein (HDL) cholesterol, beneficial for cardiovascular health (29), potentially contributing to the improvement in the atherogenic index observed in the intervention group.

Different studies have reported varying results regarding the relationship between low-carbohydrate diets and anthropometric parameters associated with metabolic syndrome. For instance, the study conducted by Sangsefidi et al. (30) demonstrated a significant inverse relationship between a low-carbohydrate diet and abdominal obesity, specifically in men. Brinkworth et al. (31) also found that a low-carbohydrate diet could reduce abdominal fat mass by approximately 30%. Abdominal obesity or abdominal fat mass is often assessed using waist-to-hip ratio measurements, which provide a more specific indication of central adiposity (32). Given that the waist-to-hip ratio did not yield significance in our study, it appears that these findings contradict one another. The contradiction between these findings and our study could result from various factors. These include differences in sample characteristics, study design, statistical power, confounding variables, publication bias, and chance. Additionally, it is worth noting that these studies highlight the significance of low-carb diets, which may resonate with the outcomes of our study.

In line with the results of the present study, Cucuzzella et al. (33) indicated that a low-carbohydrate diet led to weight loss and waist circumference reduction, decreased hunger, and increased energy levels. Mousavi et al. (34) also demonstrated that a low-carbohydrate diet significantly reduced weight, BMI and waist circumference. In this study, alongside the parameters previously mentioned, researchers also observed a reduction in hip circumference following the intervention, a contrast to our study’s findings. This difference in results between the two studies could be attributed to various factors such as differences in the study population, methodology, duration of the intervention, or even variations in the dietary protocols followed.

However, in contrast to the findings of the present study, a cross-sectional study conducted by Jafari-Maram et al. (35) in Iranian women revealed no significant relationship between a low-carbohydrate diet and obesity. Similarly, a meta-analysis reported that carbohydrate intake had no impact on the risk of obesity (36). Ha et al. (37) also found no association between a low-carbohydrate diet and metabolic syndrome, including waist circumference. Different findings among the studies can be attributed to several factors. Differences in study design, methodology, sample characteristics, and measurement tools can contribute to the disparities observed. Additionally, the percentage of carbohydrates used in the studies may have varied, potentially underestimating its effect on the desired parameters. Furthermore, people in different countries have diverse lifestyles and eating habits, which can impact the results of the studies. Additionally, the researchers should not overlook the influence of medications and medical conditions that can interfere with weight loss and size reduction.

The results of the present study revealed a significant improvement in total cholesterol, triglycerides, HDL levels, FBS, and HbA1c in the intervention group following the low-carbohydrate diet. Conversely, the control group exhibited fewer changes in these parameters. LDL levels significantly differed before and after intervention between groups. However, LDL did not differ significantly between groups.

These findings can be attributed to several factors. Implementing a low-carbohydrate diet emphasizing reduced carbohydrate intake and increased protein consumption, healthy fats, and non-starchy vegetables positively impacted these variables. By reducing the consumption of simple carbohydrates, which are known to have adverse effects on blood lipids and insulin resistance, a low-carbohydrate diet can help improve these parameters. Furthermore, increasing the intake of healthy fats can contribute to a favorable cholesterol profile by elevating HDL levels (38). Additionally, carbohydrate restriction has the potential to enhance insulin sensitivity, leading to improved blood sugar control and reduced HbA1c levels (39). Furthermore, weight loss and changes in body composition associated with low-carbohydrate diets can indirectly influence lipid profiles and blood sugar control (40).

Several studies conducted in different countries have highlighted the significant role of a low-carbohydrate diet in improving indicators of metabolic syndrome. For instance, studies conducted in Korea have demonstrated a positive association between white rice consumption, elevated fasting triglyceride and glucose levels, low HDL-C levels, and the increasing risk of metabolic syndrome (41, 42). Ha et al. (37) also found that a low-carbohydrate diet was significantly associated with a reduced risk of dyslipidemia among Korean adults, potentially due to increased HDL-C levels.

Moreover, previous randomized controlled trials (RCTs) have indicated that a low-carbohydrate diet can improve HDL-C levels (20, 43). A recent cross-sectional study in Japan discovered a positive association between a low-carbohydrate diet and HDL-C levels, regardless of the food source (44). Considering that high carbohydrate intake is a recognized risk factor for decreased HDL-C levels, a low-carbohydrate diet demonstrates a clear inverse association with dyslipidemia among Korean adults consuming a high-carbohydrate diet (37). In Iran, the study conducted by Sangsefidi et al. (30) demonstrated a significant relationship between a low-carbohydrate diet and low HDL-C levels in all participants. Similarly, a study by Mousavi et al. (34) in Iran showed that a low-carbohydrate diet significantly reduced serum triglycerides and increased HDL-C levels.

Systematic review studies have further supported the negative association between a low-carbohydrate diet, high blood pressure, and low HDL-C levels (25, 45). The results of a meta-analysis conducted by Lei et al. (22) also indicated that individuals following a low-carbohydrate diet experienced a significant decrease in triglyceride levels, diastolic blood pressure, and weight, as well as a significant increase in HDL-C level.

Studies conducted in Western countries have also reported improved weight loss and lipid profiles, including triglyceride and HDL cholesterol levels, among individuals adhering to a low-carbohydrate diet (20, 43). Additionally, a low-carbohydrate diet has shown effectiveness in managing type 2 diabetes among Australian adults (46, 47). Cucuzzella et al. (33) indicated that most participants experienced improvements in HbA1c levels, blood glucose measurements, and lipid panel results.

In contrast to our findings, certain studies conducted among Iranian adults have reported no significant association between HDL cholesterol and a low-carbohydrate diet (48, 49). Several factors may contribute to the disparity between the results of these studies and the present study, such as differences in study design, the proportion of carbohydrates used, and the study population.

Despite the methodological strengths of this multi-stage, controlled study, several limitations warrant consideration. One of the limitations is the small sample size, which restricts the generalizability of the intervention’s effects to a broader population. The study’s intervention duration, which was limited to 3 months, may not sufficiently capture dietary changes’ long-term effects and sustainability on metabolic syndrome patients. The high cost of investigations and diagnostic devices and time limitations impede the ability to measure the long-term outcome for the study group. Reliance on self-reported dietary adherence could introduce bias or inaccuracies in reporting. Finally, lack of National publication resources to support the findings of the present study.

### 4.1 Conclusion

The study results suggest that implementing nurse-led low-carbohydrate dietary interventions may lead to significant improvements in critical anthropometric and metabolic parameters among patients with metabolic syndrome. This finding highlights the potential for nurses to play a crucial role in promoting metabolic health through dietary education and support. Nurses can implement the study findings by educating and supporting patients in adopting low-carbohydrate diets effectively. Strategies include personalized counseling, education on carbohydrate sources, meal planning assistance, behavioral support, regular follow-up, multidisciplinary collaboration, and patient empowerment. By empowering patients with knowledge and skills, nurses can facilitate successful adoption and maintenance of low-carbohydrate diets among those with metabolic syndrome, improving health outcomes and quality of life. However, further research is needed to explore such interventions’ long-term effects, mechanisms, and optimal carbohydrate thresholds, emphasizing interdisciplinary collaboration among healthcare professionals. Engaging in Randomized Controlled Trials (RCTs) featuring extended follow-up periods to evaluate sustained effects, conducting Prospective Cohort Studies to explore correlations between dietary patterns and metabolic health outcomes, undertaking Meta-Analyses and Systematic Reviews to synthesize evidence and pinpoint research gaps, exploring Intervention Studies within specific population subgroups to customize interventions to varied needs, and implementing Community-Based Interventions to foster real-world behavior change, exemplify these research approaches. These studies should focus on populations with a high prevalence of metabolic syndrome and be conducted across various settings to ensure broad applicability and significant public health impact. Overall, integrating these findings into clinical practice and research could advance strategies for effectively managing and preventing metabolic syndrome.

## Data availability statement

The original contributions presented in the study are included in the article/supplementary material, further inquiries can be directed to the corresponding author.

## Ethics statement

This research was conducted after obtaining the institutional review board agreement numbered Ref: 1540 on May 8th, 2023, from Baghdad University. The studies were conducted in accordance with the local legislation and institutional requirements. The participants provided their written informed consent to participate in this study.

## Author contributions

MA: Writing – original draft, Writing – review & editing. SA-F: Writing – original draft, Writing – review & editing.
